# Dietary inadequacies and their association with pain severity and disc degeneration in patients surgically treated for degenerative lumbosacral spine disorders

**DOI:** 10.3389/fnut.2026.1910742

**Published:** 2026-07-30

**Authors:** Damian Strojny, Tomasz Kulpok, Irena Puszkarz, Tomasz Hendzel, Małgorzata Soroń-Lisik, Rafał Staszkiewicz, Dawid Sobański, Beniamin Oskar Grabarek

**Affiliations:** 1 Collegium Medicum, WSB University, Dabrowa Gornicza, Poland; 2 Department of Neurology, New Medical Techniques Specjalist Hospital of St. Family in Rudna Mała, Rudna Mala, Poland; 3 Medical Care Center “Kopernik” Warsaw, Warsaw, Poland; 4 Doctoral School, University of Rzeszow, Rzeszow, Poland; 5 Department of Neurosurgery, 5th Military Clinical Hospital with the SP ZOZ Polyclinic in Krakow, Krakow, Poland; 6 Department of Neurosurgery, Faculty of Medicine in Zabrze, Academy of Silesia, Katowice, Poland; 7 Department of Neurosurgery, Szpital sw. Rafala in Cracow, Cracow, Poland

**Keywords:** dietary intake, inadequate vitamin D intake, intervertebral disc degeneration, low back pain, lumbar spinal stenosis, musculoskeletal nutrition, nutritional status, omega-3 fatty acids

## Abstract

**Background:**

Intervertebral disc degeneration (IVDD) and lumbar spinal stenosis (LSS) are leading causes of musculoskeletal disability, and dietary factors are increasingly implicated in their progression, yet nutritional profiling in surgically treated patients remains scarce.

**Objective:**

To evaluate dietary intake and nutrient inadequacies in surgical spine patients and their associations with clinical outcomes.

**Design:**

Cross-sectional study of 200 IVDD (microdiscectomy) and 180 LSS (hemilaminectomy) patients. Dietary intake was assessed using repeated 24-h recalls and compared with Estimated Energy Requirement (EER), Estimated Average Requirement (EAR), and Adequate Intake (AI) reference values. Associations between nutrient intake and pain severity, measured with the Visual Analog Scale (VAS), disc degeneration (Pfirrmann grade), and, in the LSS cohort, radiological stenosis severity were examined using multivariable regression models in which all candidate nutrients were entered simultaneously with demographic, anthropometric, and lifestyle covariates (age, sex, body mass index (BMI), energy intake, smoking, diabetes, alcohol use, occupation, education), with false discovery rate (FDR) correction for multiple comparisons.

**Results:**

Both cohorts showed consistent, pronounced dietary inadequacies. Over 80% of participants had insufficient eicosapentaenoic acid (EPA) and docosahexaenoic acid (DHA) intake, up to 95% of men had inadequate fiber intake, and over 90% did not meet recommended vitamin D intake. Approximately 70% had inadequate folate intake, and calcium intake was below recommended levels in most patients across subgroups. In simultaneous multivariable models adjusted for the full covariate panel, no nutrient retained a significant independent association with VAS, Pfirrmann grade, or radiological stenosis severity after FDR correction; nominal, uncorrected associations were observed for magnesium and zinc with pain severity in the LSS cohort only.

**Conclusion:**

Surgically treated spine patients show widespread inadequate intake of several nutrients relevant to musculoskeletal and connective tissue health, most notably vitamin D, calcium, fiber, and omega-3 fatty acids. Although independent associations with pain and radiological severity were not confirmed after adjustment, the high prevalence of dietary inadequacy supports consideration of routine nutritional screening, and prospective studies incorporating serum biomarkers are warranted to clarify whether a causal relationship exists.

## 1 Introduction

Degenerative disorders of the lumbosacral spine are among the most common causes of chronic musculoskeletal disability worldwide, with substantial clinical and socioeconomic burden (1, 2). Intervertebral disc degeneration (IVDD) and lumbar spinal stenosis (LSS) are particularly prevalent in aging populations and major contributors to low back pain, neurogenic claudication, and functional impairment (3). These disorders arise from multifactorial processes involving mechanical overload, structural degeneration, inflammatory signaling, and metabolic alterations within the disc and surrounding structures (4).

The pathogenesis of degenerative spinal disease involves progressive deterioration of the extracellular matrix, including loss of proteoglycans, disc dehydration, collagen disruption, and hypertrophy of supporting structures such as the ligamentum flavum (5–7). In LSS, ligamentum flavum thickening together with disc degeneration and facet joint changes narrows the spinal canal and compresses neural elements, accompanied by inflammatory activation, oxidative stress, and altered cellular metabolism that further accelerate tissue degeneration (8, 9).

Although mechanical and genetic factors are well recognized in the development of degenerative spinal diseases, increasing evidence suggests that systemic metabolic conditions and lifestyle-related factors also play an important role in their progression (10, 11). Obesity, metabolic syndrome, and chronic low-grade inflammation have been associated with accelerated IVDD and increased severity of spinal pathology (12, 13). In this context, nutritional status and dietary habits represent potentially modifiable determinants that may influence both structural degeneration and clinical manifestations of spinal disorders (14–16).

The intervertebral disc is the largest avascular structure in the human body, comprising a central, proteoglycan-rich nucleus pulposus surrounded by the collagenous annulus fibrosus and bounded superiorly and inferiorly by the cartilaginous endplates. Because the mature disc lacks a direct blood supply, disc cells depend almost entirely on diffusion of oxygen, glucose, and other nutrients from capillaries in the vertebral body, across the cartilaginous endplate, and through the dense extracellular matrix to reach cells in the nucleus pulposus (17). This diffusion pathway is long relative to other tissues and highly sensitive to endplate calcification, matrix density, and systemic nutrient availability; reduced diffusive capacity with aging and degeneration is thought to contribute to a hostile microenvironment characterized by low glucose, low pH, and hypoxia, which further impairs disc cell viability and matrix synthesis (18). This unique reliance on diffusive, systemically dependent nutrient delivery provides a direct physiological rationale for why circulating and dietary nutrient availability may be particularly consequential for disc health, in contrast to well-vascularized musculoskeletal tissues.

Adequate dietary intake of macronutrients, vitamins, and minerals is essential for maintaining the structural integrity and metabolic activity of musculoskeletal tissue (19, 20). Several nutrients participate in connective tissue homeostasis, matrix remodeling, and inflammatory regulation (19, 20). Omega-3 fatty acids, in particular, exhibit anti-inflammatory properties (21), while magnesium (Mg), zinc (Zn), and vitamin D support bone metabolism and antioxidant defense (22, 23). Conversely, nutritional inadequacies may promote inflammation and metabolic dysregulation, potentially exacerbating spinal tissue degeneration (14–16).

Despite growing interest in the relationship between nutrition and musculoskeletal health, relatively few studies have comprehensively evaluated dietary patterns and nutrient intake in patients with degenerative spinal disorders. Existing research has primarily focused on isolated nutrients or metabolic comorbidities rather than on the broader nutritional profile of affected individuals. Furthermore, the potential associations between dietary inadequacies and clinical parameters such as pain severity or structural degeneration remain insufficiently characterized (24–26).

Therefore, the aim of the present study was to **e**valuate the nutritional status and dietary intake patterns in patients undergoing surgical treatment for degenerative lumbosacral spine disorders, including IVDD and LSS. Particular attention was given to the intake of energy, macronutrients, vitamins, and mineral components, as well as to the prevalence of nutrient inadequacies.

## 2 Materials and methods

This study methodology was built upon the work conducted in our previous papers (23, 27–29).

This study was approved by the Bioethics Committee of the State Academy of Applied Sciences in Przemyśl (approval no. 8/2024, issued on August 1, 2024) and by the Bioethics Committee at the District Medical Chamber in Kraków (approval no. 224/KBL/OIL/2022, issued on December 16, 2022; no. 162/KBL/OIL/2021, June 11, 2021). All procedures were conducted in accordance with the principles outlined in the 2013 Declaration of Helsinki. Written informed consent was obtained from all living participants prior to inclusion in the study, and patient data were pseudonymized to ensure privacy and confidentiality.

Data completeness was verified prior to analysis. Missing data were infrequent and did not exceed 2.3% for any analyzed variable, limited primarily to isolated dietary recall items or incomplete questionnaire responses; demographic, anthropometric, clinical, and radiological variables were complete. Completed questionnaires were reviewed immediately after collection to identify missing or inconsistent responses, which were clarified directly with participants whenever possible. Because missingness was infrequent and no systematic pattern was identified, an available-case approach was used without imputation, and the number of observations included in each analysis is reported in the corresponding tables.

### 2.1 Study population

The study included two independent cohorts of patients undergoing surgical treatment for degenerative disorders of the lumbosacral spine. The first group consisted of patients with degenerative IVDD undergoing lumbar microdiscectomy, while the second group included patients with LSS associated with hypertrophy of the ligamentum flavum treated with decompressive hemilaminectomy.

The IVDD cohort comprised 200 Caucasian patients (94 women and 106 men) with a mean age of 49.56 ± 15.19 years. These patients were diagnosed with degenerative pathology of the lumbosacral intervertebral disc and were qualified for lumbar microdiscectomy. The analyzed cohort has been partially characterized in previous studies conducted by our research group.

The LSS cohort consisted of 180 patients presenting with degenerative lumbar spinal stenosis accompanied by hypertrophy of the ligamentum flavum. This group included 87 women and 93 men with a mean age of 51.12 ± 2.98 years. All participants met standard neurosurgical criteria for decompressive surgery and underwent hemilaminectomy performed by an experienced neurosurgeon.

All patients were adults aged 18 years or older and provided written informed consent prior to inclusion in the study.

Anthropometric measurements, including body weight and height, were recorded for all participants and used to calculate body mass index (BMI). According to the World Health Organization classification, individuals were categorized as having normal weight (BMI < 25 kg/m^2^), overweight (25–29.9 kg/m^2^), or obesity (≥30 kg/m^2^).

Participants were additionally interviewed regarding lifestyle factors. All individuals confirmed that during the 6 months preceding inclusion they had not followed any special diet and had not used vitamin or mineral supplements classified as medicinal preparations. None of the patients reported occupational or accidental exposure to heavy metals.

Baseline demographic and anthropometric characteristics of both study groups are summarized in Table 1.

**Table 1:** Demographic and anthropometric characteristics of the analyzed cohorts.

Parameter	IVDD patients (*n* = 200)	LSS patients (*n* = 180)
Age (years, mean ± SD)	49.56 ± 15.19	51.12 ± 2.98
Sex (female/male)	94/106	87/93
BMI (kg/m^2^, mean ± SD)	26.43 ± 3.38	27.8 ± 3.9
Normal weight (<25)	43 (21.5%)	42 (23.3%)
Overweight (25–29.9)	105 (52.5%)	78 (43.3%)
Obese (≥30)	52 (26.0%)	60 (33.3%)

### 2.2 Clinical evaluation

All patients underwent a comprehensive neurological examination performed by the same neurosurgeon according to a standardized protocol in order to minimize interobserver variability. The clinical assessment included evaluation of muscle strength and tone in the lower extremities, passive range of motion, deep tendon reflexes (patellar and Achilles), and pathological reflexes such as plantar, Babinski, and Rossolimo responses. In addition, sensory perception, gait pattern, posture, spinal mobility, and palpation of the lumbosacral region were systematically assessed. Clinical findings were recorded in a dedicated study database.

### 2.3 Pain assessment

Pain severity was quantified using the Visual Analog Scale (VAS), which ranges from 0 (no pain) to 10 (maximum perceived pain intensity). In the IVDD cohort, reported VAS scores ranged from 3 to 10 points, with the highest frequencies observed for scores of 3 and 5. In the LSS cohort, all patients reported VAS scores between 4 and 10. The distribution of pain severity in the LSS group showed that 24 patients reported a VAS score of 4, 42 patients reported a score of 5, and the largest subgroup consisted of 51 individuals with a score of 6. Higher pain levels were less frequent, with 27 patients reporting a score of 7, while scores of 8, 9, and 10 were each observed in 12 patients.

The distribution of pain intensity in both cohorts is presented in Table 2.

**Table 2:** Distribution of pain intensity assessed using the VAS scale.

VAS score	IVDD patients (*n* = 200)	LSS patients (*n* = 180)
3	54	–
4	28	24
5	41	42
6	32	51
7	8	27
8	20	12
9	6	12
10	11	12

VAS, Visual Analog Scale.

### 2.4 Magnetic resonance imaging

Preoperative magnetic resonance imaging was performed for all patients as part of the standard diagnostic work-up and was reported by a radiologist in routine clinical practice to confirm degenerative pathology of the lumbosacral spine. Imaging was conducted using scanners with a magnetic field strength of at least 1.5 Tesla. The imaging protocol included spin echo T1-weighted sequences, T1-weighted FLAIR imaging, fast spin echo T2-weighted sequences, and Short Tau Inversion Recovery (STIR) sequences. Images were obtained in sagittal and axial planes with slice thicknesses of 3 mm and 4 mm to ensure accurate visualization of intervertebral disks, spinal canal structures, and ligamentum flavum morphology.

Independently of the routine radiological report, and separately for each cohort, the same MRI examinations were additionally reviewed for study purposes by two experienced neurosurgeons. In the IVDD cohort, both observers assessed disc degeneration using the Pfirrmann classification (weighted Cohen’s *κ* = 0.84); in the LSS cohort, both observers assessed the radiological stenosis severity parameters described in the Statistical Analysis section (weighted Cohen’s κ = 0.83). Discrepant gradings were resolved by consensus following joint review.

### 2.5 Assessment of intervertebral disc degeneration

In the IVDD cohort, the severity of intervertebral disc degeneration was evaluated using the Pfirrmann classification system based on MRI findings. The grading was independently performed by two experienced neurosurgeons. Among the analyzed patients, grade 4 degeneration was the most frequent and was observed in 117 individuals (58.5%). Grade 3 degeneration was identified in 45 patients (22.5%), grade 2 changes in 32 individuals (16.0%), and grade 5 degeneration in 6 patients (3.0%).

### 2.6 Dietary assessment

Dietary intake was evaluated using a structured questionnaire based on the 24-h dietary recall method, implemented in accordance with recommendations of the Committee on Human Nutrition Science of the Polish Academy of Sciences (30). To improve the reliability of the dietary assessment and better reflect habitual intake, three separate recalls were collected from each participant: two referring to weekdays and one to a weekend day. This approach helps minimize intra-individual variation in daily dietary patterns.

Portion sizes were estimated using the Album of Photographs of Food Products and Dishes developed by the National Food and Nutrition Institute in Warsaw (31), which facilitates standardized estimation of consumed quantities. Nutritional composition and energy values of the reported diets were calculated using Dieta 6.0 software (National Food and Nutrition Institute, Warsaw, Poland), based on data derived from the Tables of Composition and Nutritional Value of Foods (32).

Dietary assessment was performed during the preoperative evaluation, prior to surgical treatment, and was administered by trained members of the research team using the standardized recall protocol described above. The dietary assessment protocol followed nationally recommended methodology and employed standardized, previously validated assessment tools; however, no additional validation of the dietary recalls was performed specifically within this study cohort, which is acknowledged as a limitation.

Anthropometric measurements, including body weight and height, were obtained for all participants, and BMI was calculated. The estimated daily energy intake was subsequently analyzed in relation to BMI categories. Mean daily energy consumption and macronutrient intake were determined from the collected dietary recalls and compared with appropriate dietary reference standards. These included Estimated Energy Requirement (EER), Estimated Average Requirement (EAR), and Adequate Intake (AI) values according to the Dietary Reference Intakes established by the U. S. Institute of Medicine (National Academies of Sciences, Engineering, and Medicine) (33). In addition, the proportion of individuals meeting or failing to meet recommended intake levels was calculated, together with the percentage contribution of protein, fat, and carbohydrates to total daily energy intake.

### 2.7 Mineral intake

Dietary intake of selected minerals and trace elements was also evaluated. The analyzed macronutrients included sodium (Na), potassium (K), calcium (Ca), phosphorus (P), and Mg, while trace elements comprised iron (Fe), Zn, copper (Cu), manganese (Mn), and iodine (I). Intake levels were compared with established dietary reference values. For sodium and potassium, intake was assessed relative to Adequate Intake (AI) recommendations, whereas for the remaining elements comparisons were made using Estimated Average Requirement (EAR) values.

For nutrients with defined EAR thresholds, the cut-point method was applied to estimate the prevalence of inadequate intake within the study population. In the case of sodium, potassium, and manganese, the proportion of individuals meeting the AI recommendations was determined. Because national Polish dietary guidelines do not specify recommended manganese intake, reference values proposed by the Institute of Medicine of the National Academies (USA) were adopted. According to these guidelines, the recommended intake for adults aged 19 years or older is 1.8 mg/day for women and 2.3 mg/day for men (34).

### 2.8 Vitamin intake

The dietary intake of several vitamins was analyzed, including vitamins A, E, D, B₁ (thiamine), B₂ (riboflavin), niacin, B₆, B₁₂, folate, and vitamin C. For vitamins D and E, intake levels were evaluated relative to Adequate Intake (AI) values, while the intake of the remaining vitamins was compared with Estimated Average Requirement (EAR) recommendations. Based on these comparisons, the prevalence of both adequate and inadequate vitamin intake within the study population was calculated.

### 2.9 Food frequency assessment

In addition to the quantitative dietary assessment, participants completed a food frequency questionnaire (FFQ) developed by the Committee on Human Nutrition Science of the Polish Academy of Sciences, covering 37 groups of food products (33). Participants reported how often specific foods were consumed during a typical week. Based on the reported frequency, food items were classified into two categories. The first category represented infrequent consumption, defined as intake once per week or less. The second category represented frequent consumption, defined as intake at least two to three times per week. For fresh and canned fish, frequent consumption was defined as intake at least once per week.

Certain products, including wine, vodka, and canned meat, were excluded from further analysis because all participants reported very rare consumption of these items. A detailed description of the food groups, example products, and classification criteria is provided in Supplementary Table S1.

For analytical purposes, the consumption categories were operationalized as Group I (infrequent consumption; ≤1 time per week) and Group II (frequent consumption; ≥2–3 times per week, or ≥1 time per week for fish). Consequently, the results reported for Group I and Group II correspond to patients characterized by relatively low versus high intake frequency of the analyzed food categories, enabling assessment of potential associations between dietary patterns, disease severity, and therapeutic response.

### 2.10 Statistical analysis

Statistical analyses were performed using StatPlus version 1.1 (AnalystSoft Inc., Brandon, FL, United States). Statistical analyses were performed separately for the IVDD and LSS cohorts, with additional pooled analyses where appropriate for nutrient inadequacy and clinical outcome modeling. Continuous variables were expressed as mean ± standard deviation (SD), whereas categorical variables were presented as counts and percentages with 95% confidence intervals (95% CIs).

The distribution of continuous variables was assessed using the Shapiro–Wilk test. Since the analyzed variables met the assumptions of normality, comparisons of mean nutrient, vitamin, and mineral intake between women and men within each study group were performed using the independent-samples Student’s t-test. A two-sided *p* value of less than 0.05 was considered statistically significant.

The prevalence of inadequate intake was calculated separately for women and men in both cohorts for macronutrients, selected fatty acids, vitamins, and mineral components. Inadequacy was defined according to the appropriate dietary reference value, including estimated average requirement (EAR), adequate intake (AI), or estimated energy requirement (EER), depending on the nutrient analyzed. Proportions were reported as percentages with corresponding 95% confidence intervals.

To evaluate the associations between nutrient intake and clinical or radiological severity, multivariable linear regression models were constructed. For pain severity analyses, Visual Analog Scale (VAS) score was used as the dependent variable, and separate models were fitted for the IVDD and LSS cohorts. For structural degeneration analyses, Pfirrmann grade was used as the dependent variable and models were restricted to the IVDD cohort; in the LSS cohort, the same modeling strategy was additionally applied to radiological markers of stenosis severity obtained from preoperative MRI (Schizas grade, ligamentum flavum thickness, anteroposterior canal diameter, dural sac cross-sectional area, and central, facet, foraminal, and lateral recess stenosis grades). The seven nutrients most frequently characterized by inadequate intake in the descriptive analysis (EPA + DHA, fiber, folate, magnesium, vitamin D, calcium, and zinc) were entered simultaneously as continuous, standardized (z-scored) predictors in each model, rather than individually, in order to account for their mutual correlation and to avoid inflation of false-positive associations from testing each nutrient in a separate model. Variance inflation factors (VIFs) were calculated for all predictors to formally assess multicollinearity. Each model was additionally adjusted for age, sex, BMI, total daily energy intake, smoking status, diabetes, alcohol consumption, occupation type (physical versus sedentary/mental work, used as a proxy for habitual physical activity), and educational attainment (used as a proxy for socioeconomic status). Data on physical activity level, medication use (including nonsteroidal anti-inflammatory drugs [NSAIDs] and corticosteroids), comorbid inflammatory diseases, and depression or anxiety status were not collected in this cohort and, therefore, could not be included in the analyses. This limitation is acknowledged in the Discussion as a potential source of residual confounding. Overall model fit was assessed using R^2^ and adjusted R^2^, and overall model significance using the F-test.

All regression models were adjusted for body mass index (BMI), age, sex, and total daily energy intake. Effect estimates were expressed as *β* coefficients with 95% confidence intervals and two-sided *p* values. To account for multiple comparisons across nutrient-specific models within each outcome family, false discovery rate (FDR) correction was applied using the Benjamini–Hochberg procedure, and adjusted q values were reported alongside raw p values.

All statistical tests were two-tailed. The level of statistical significance was set at *p* < 0.05, while associations remaining significant after FDR correction were interpreted based on q < 0.05.

#### 2.10.1 Sample size justification

For the IVDD cohort, sample size was estimated using population-based prevalence data. According to the Polish Central Statistical Office (2019), the adult population of Poland was 38,383,000; using the sample-size calculator available at naukowiec.org/dobor.html (accessed 21 March 2020) (35), a maximum error of 9% at *p* < 0.05 indicated a minimum requirement of 119 participants for the general population estimate. Using the 2019 European Health Survey estimate that 25.8% of Polish adults (*n* = 31,435,677) report lumbosacral pain, the same error margin yielded a minimum requirement of 100 participants (36). The recruited IVDD cohort (n = 200) exceeded both thresholds. For the LSS cohort, sample size was calculated using G*Power 3.1 (37) based on effect sizes reported in prior studies of elemental composition in degenerative spinal disease; a minimum of 150 patients and 100 controls was required to achieve 80% power (*β* = 0.20) at *α* = 0.05. The recruited LSS cohort (180 patients) exceeded this threshold, and Cohen’s d was subsequently used to contextualize the clinical relevance of key elemental differences.

## 3 Results

### 3.1 Energy and macronutrient intake in IVDD and LSS patients

The mean daily energy intake in patients with IVDD was lower than the estimated energy requirement in both sexes (Table 3). Despite the reduced overall energy intake, the macronutrient distribution indicated an imbalanced dietary pattern characterized by a relatively high contribution of dietary fat and a reduced proportion of carbohydrates.

**Table 3:** Estimated energy requirements, estimated average requirements, and adequate intake standards for energy and nutrients in men and women with IVDD.

Energy and nutrients	Gender	Mean ± SD	Norm	Quantity/day	*p*-value *t*-student’s test
Energy [kcal]	Women (*n* = 94)	1820.40 ± 248.60	EER	2142.00 ± 181.50	<0.05
	Men (*n* = 106)	2352.80 ± 326.40		2724.00 ± 236.80	
% energy from protein	Women	16.8 ± 3.4	–	10–15	>0.05
	Men	15.2 ± 3.1		10–15	
% energy from fat	Women	34.6 ± 6.2	–	20–35	<0.05
	Men	35.1 ± 7.0		20–35	
% energy from carbohydrates	Women	48.8 ± 7.5	–	50–70	>0.05
	Men	49.7 ± 8.1		50–70	
Protein [g]	Women	76.3 ± 13.2	EAR	52.4 ± 5.1	<0.05
	Men	89.1 ± 16.0		66.3 ± 7.0	
Animal protein [g]	Women	49.8 ± 10.9	–	–	<0.05
	Men	61.9 ± 14.6			
Plant protein [g]	Women	26.5 ± 7.3	–	–	>0.05
	Men	27.2 ± 8.1			
Fat [g]	Women	70.0 ± 15.1	EAR	71.4 ± 8.3	>0.05
	Men	91.8 ± 19.4		90.8 ± 11.2	
LA [g]	Women	8.3 ± 2.8	AI	11.0	<0.05
	Men	10.7 ± 3.4		14.0	
ALA [g]	Women	1.0 ± 0.4	AI	1.1	>0.05
	Men	1.2 ± 0.5		1.4	
EPA + DHA [mg]	Women	122.6 ± 69.4	AI	250	<0.05
	Men	148.3 ± 77.2		250	<0.05
Cholesterol [g]	Women	228.4 ± 94.1	–	–	>0.05
	Men	307.6 ± 118.8			
Unsaturated fatty acids [g]	Women	26.1 ± 8.6	–	–	>0.05
	Men	31.4 ± 10.1			
Monounsaturated fatty acids [g]	Women	23.8 ± 7.4	–	–	>0.05
	Men	27.9 ± 8.0			
Polyunsaturated fatty acids [g]	Women	9.1 ± 3.1	–	–	>0.05
	Men	10.9 ± 3.8			
Carbohydrates [g]	Women	222.0 ± 58.6	–	–	>0.05
	Men	292.0 ± 74.2			
Digestible carbohydrates [g]	Women	198.7 ± 52.8	AI	100	<0.05
	Men	262.8 ± 66.4			
Fiber [g]	Women	23.3 ± 7.1	AI	25	>0.05
	Men	21.4 ± 6.3			

Data are presented as mean ± standard deviation (SD). Standards are reported as estimated energy requirements (EER), estimated average requirements (EAR), or adequate intake (AI), where applicable. Comparisons between sexes were conducted using the independent-samples Student’s t-test. Data distribution was verified using the Shapiro–Wilk test, confirming normality assumptions. LA, linoleic acid; ALA, alpha-linolenic acid; EPA, eicosapentaenoic acid; DHA, docosahexaenoic acid; EER, estimated energy requirements; EAR, estimated average requirements; AI, adequate intake; kcal, kilocalorie; g, gram; mg, milligram.

Protein intake exceeded the estimated average requirement in both women and men, with a predominance of animal-derived protein sources. In contrast, the intake of essential fatty acids showed notable inadequacies. Linoleic acid intake remained below the recommended adequate intake in both sexes, while the consumption of long-chain omega-3 fatty acids (EPA + DHA) was markedly insufficient relative to recommended levels.

Total fat intake approximated the recommended levels; however, the distribution of fatty acids suggested an unfavorable lipid profile, with relatively high cholesterol intake and moderate consumption of mono- and polyunsaturated fatty acids. Carbohydrate intake was within expected ranges overall, although digestible carbohydrate consumption exceeded the reference value.

Dietary fiber intake approached the recommended adequate intake in women but remained below recommended levels in men. Collectively, these findings indicate that although total protein intake was adequate or elevated, the overall dietary pattern in IVDD patients was characterized by insufficient energy intake, excessive reliance on animal protein, suboptimal intake of essential fatty acids, and inadequate omega-3 fatty acid consumption.

In patients with LSS, mean daily energy intake remained below the estimated energy requirement in both women and men (Table 4). The macronutrient distribution indicated a dietary pattern characterized by an elevated proportion of energy derived from fat and a relatively reduced contribution from carbohydrates.

**Table 4:** Estimated energy requirements, estimated average requirements, and adequate intake standards for energy and nutrients in men and women.

Energy and nutrients	Gender	Mean ± SD	Norm	Quantity/day	*p*-value *t*-student’s test
Energy [kcal]	Women	1742.60 ± 221.40	EER	2098.00 ± 166.20	<0.05
	Men	2241.30 ± 294.70		2662.00 ± 214.50	
% energy from protein	Women	17.0 ± 3.6	–	10–15	>0.05
	Men	15.4 ± 3.3		10–15	
% energy from fat	Women	35.1 ± 6.8	–	20–35	<0.05
	Men	37.8 ± 7.5		20–35	
% energy from carbohydrates	Women	46.9 ± 7.4	–	50–70	<0.05
	Men	44.6 ± 8.0		50–70	
Protein [g]	Women	74.1 ± 12.6	EAR	54.1 ± 5.4	<0.05
	Men	86.1 ± 15.3		68.4 ± 7.4	
Animal protein [g]	Women	50.6 ± 11.2	–	–	<0.05
	Men	63.4 ± 13.8		–	
Plant protein [g]	Women	23.5 ± 6.9	–	–	>0.05
	Men	22.7 ± 7.6		–	
Fat [g]	Women	68.0 ± 14.7	EAR	69.9 ± 7.6	<0.05
	Men	94.1 ± 20.1		88.7 ± 9.8	
LA [g]	Women	7.9 ± 2.5	AI	11.0	<0.05
	Men	10.1 ± 3.1		14.0	
ALA [g]	Women	0.9 ± 0.3	AI	1.1	<0.05
	Men	1.0 ± 0.4		1.4	
EPA + DHA [mg]	Women	101.8 ± 61.7	AI	250	<0.05
	Men	129.4 ± 70.2		250	
Cholesterol [g]	Women	239.7 ± 96.8	–	–	>0.05
	Men	326.2 ± 121.4		–	
Unsaturated fatty acids [g]	Women	24.9 ± 8.0	–	–	>0.05
	Men	29.8 ± 9.4		–	
Monounsaturated fatty acids [g]	Women	22.6 ± 7.0	–	–	>0.05
	Men	27.6 ± 7.8		–	
Polyunsaturated fatty acids [g]	Women	8.4 ± 2.8	–	–	>0.05
	Men	9.8 ± 3.4		–	
Carbohydrates [g]	Women	204.3 ± 54.1	–	–	<0.05
	Men	249.8 ± 67.5		–	
Digestible carbohydrates [g]	Women	181.7 ± 48.0	AI	100	<0.05
	Men	223.5 ± 60.4		100	
Fiber [g]	Women	18.9 ± 5.9	AI	25	<0.05
	Men	17.2 ± 5.4		25	

Data are presented as mean ± standard deviation (SD). Standards are reported as estimated energy requirements (EER), estimated average requirements (EAR), or adequate intake (AI), where applicable. Comparisons between sexes were conducted using the independent-samples Student’s t-test. Data distribution was verified using the Shapiro–Wilk test, confirming normality assumptions. LA, linoleic acid; ALA, alpha-linolenic acid; EPA, eicosapentaenoic acid; DHA, docosahexaenoic acid; EER, estimated energy requirements; EAR, estimated average requirements; AI, adequate intake; kcal, kilocalorie; g, gram; mg, milligram.

Protein intake exceeded the estimated average requirement in both sexes, with a predominance of animal-derived protein sources. Plant protein intake did not differ substantially between women and men and represented a smaller fraction of total protein consumption. Total fat intake approached recommended levels; however, the percentage of energy derived from fat slightly exceeded the recommended range, particularly in men.

The intake of essential fatty acids revealed several inadequacies. Both linoleic acid and alpha-linolenic acid intake were below the recommended adequate intake, especially in women. In addition, consumption of long-chain omega-3 fatty acids (EPA + DHA) was markedly insufficient in both sexes compared with recommended values.

Carbohydrate intake showed moderate differences between sexes, with men consuming significantly greater amounts than women. Digestible carbohydrate intake exceeded the reference value in both groups. In contrast, dietary fiber intake remained below the recommended adequate intake, particularly among men.

Overall, the dietary pattern observed in LSS patients was characterized by insufficient total energy intake, relatively high fat consumption, inadequate intake of essential fatty acids, markedly low intake of long-chain omega-3 fatty acids, and insufficient dietary fiber intake.

The prevalence analysis revealed substantial inadequacies in several macronutrients and essential fatty acids across both patient cohorts (Table 5). Insufficient dietary fiber intake was highly prevalent, particularly among men, affecting nearly nine out of 10 IVDD patients and more than 90% of men with LSS. Women in both groups also showed high rates of fiber inadequacy, although slightly lower than those observed in men.

**Table 5:** Prevalence of inadequate macronutrient and essential fatty acid intake in IVDD and LSS patients stratified by sex.

Nutrient	IVDD women (%) [95% CI]	IVDD men (%) [95% CI]	LSS women (%) [95% CI]	LSS men (%) [95% CI]
Protein	43.6 (34.0–53.7)	26.4 (19.0–35.5)	41.4 (31.6–51.9)	48.4 (38.5–58.4)
Fat	76.6 (67.1–84.0)	84.0 (75.8–89.7)	78.2 (68.4–85.5)	92.5 (85.3–96.3)
Digestible carbohydrates	28.7 (20.6–38.6)	39.6 (30.8–49.1)	25.3 (17.3–35.3)	33.3 (24.6–43.4)
Fiber	66.0 (55.9–74.7)	87.7 (80.1–92.7)	69.0 (58.6–77.7)	94.6 (88.0–97.7)
LA	71.3 (61.4–79.4)	84.9 (76.9–90.5)	85.1 (76.1–91.1)	88.2 (80.0–93.3)
ALA	73.4 (63.7–81.3)	89.6 (82.4–94.1)	71.3 (61.0–79.7)	95.7 (89.5–98.3)
EPA + DHA	90.4 (82.8–94.9)	84.0 (75.8–89.7)	90.8 (82.9–95.3)	87.1 (78.8–92.5)

LA, Linoleic Acid; ALA, Alpha-Linolenic Acid; EPA, Eicosapentaenoic Acid; DHA, Docosahexaenoic Acid.

Inadequate intake of essential fatty acids was also widespread. Inadequate intake of linoleic acid (LA) and alpha-linolenic acid (ALA) affected the majority of participants in both cohorts, with particularly high prevalence among men with LSS. The most pronounced inadequacy concerned long-chain omega-3 fatty acids (EPA + DHA), for which inadequate intake was observed in more than four-fifths of patients regardless of disease group or sex.

Fat intake inadequacy was also frequent, especially among men with LSS, where the highest prevalence was observed. Inadequate intake of digestible carbohydrates occurred less often but remained notable in both disease groups, particularly among men.

Protein inadequacy showed a more heterogeneous distribution. While it affected approximately two-fifths of women in both cohorts, the prevalence was lower among men with IVDD but higher among men with LSS.

Overall, these findings indicate that patients with degenerative spinal disorders commonly exhibit insufficient intake of dietary fiber and essential fatty acids, particularly omega-3 fatty acids, with men generally demonstrating a higher prevalence of inadequacy than women.

### 3.2 Vitamin intake and prevalence of inadequacy in IVDD and LSS patients

Analysis of vitamin intake in patients with lumbar spinal stenosis revealed several deviations from recommended dietary reference values (Table 6). Vitamin D intake was markedly below the recommended adequate intake in both women and men, indicating pronounced inadequacy of this nutrient within the LSS cohort. Folate intake was also substantially lower than the estimated average requirement in both sexes.

**Table 6:** Mean daily vitamin intake in patients with LSS compared with dietary reference values.

Vitamin	Gender	Mean ± SD	Norm		*p*-value *t*-student’s test
			Type of standard	Quantity/day	
A [μg]	Women (*n* = 87)	688.5 ± 392.8	EAR	500	<0.05
	Men (*n* = 93)	895.2 ± 349.1		630	
E [mg]	Women	8.8 ± 3.1	AI	8	<0.05
	Men	7.5 ± 2.6		10	
D [μg]	Women	2.9 ± 1.4	AI	15	<0.05
	Men	4.0 ± 1.9		15	
B1 [mg]	Women	1.08 ± 0.40	EAR	0.9	>0.05
	Men	1.30 ± 0.49		1.1	
B2 [mg]	Women	1.27 ± 0.51	EAR	0.9	<0.05
	Men	1.48 ± 0.58		1.1	
B6 [mg]	Women	1.00 ± 0.31	EAR	1.1	>0.05
	Men	1.48 ± 0.39		1.3	
B12 [μg]	Women	2.91 ± 1.05	EAR	2.0	<0.05
	Men	4.05 ± 1.14		2.0	
Niacin [mg]	Women	17.9 ± 4.6	EAR	11	<0.05
	Men	21.5 ± 4.1		12	
C [mg]	Women	68.5 ± 31.4	EAR	60	<0.05
	Men	61.4 ± 26.3		75	
Folates [μg]	Women	218.7 ± 88.3	EAR	320	<0.05
	Men	235.6 ± 81.9		320	

Data are presented as mean ± standard deviation. Data distribution was tested using the Shapiro–Wilk test; all variables met normality assumptions, and comparisons between sexes were performed with the independent-samples Student’s *t*-test. EAR, Estimated Average Requirement; AI, Adequate Intake.

In contrast, the intake of several vitamins exceeded recommended values. Mean consumption of vitamin A, vitamin B2, vitamin B12, and niacin was above the corresponding reference levels in both women and men. Vitamin C intake also exceeded the recommended level in women, while in men it remained closer to the reference value.

Vitamin E intake showed sex-specific differences, with women meeting or slightly exceeding the recommended adequate intake, whereas men remained below the recommended level. Intake of vitamin B1 and vitamin B6 generally approximated recommended values, although women exhibited slightly lower levels of vitamin B6 intake relative to the reference standard.

Overall, the vitamin intake profile in LSS patients suggests adequate or elevated consumption of several B-group vitamins and vitamin A, but persistent inadequate intake of vitamin D and folates, which represent the most pronounced micronutrient inadequacies in this cohort.

The analysis of vitamin intake in patients with IVDD revealed a pattern largely similar to that observed in the LSS cohort (Table 7). Vitamin D intake was markedly below the recommended adequate intake in both women and men, indicating pronounced inadequacy across the IVDD population. Folate intake was also substantially lower than the estimated average requirement in both sexes.

**Table 7:** Mean daily vitamin intake in patients with IVDD compared with dietary reference values.

Vitamin	Gender	Mean ± SD	Norm		*p*-value *t*-student’s test
			Type of standard	Quantity/day	
A [μg]	Women (*n* = 94)	701.6 ± 401.3	EAR	500	<0.05
	Men (*n* = 106)	908.4 ± 356.7		630	
E [mg]	Women	9.2 ± 3.2	AI	8	<0.05
	Men	7.8 ± 2.4		10	
D [μg]	Women	3.1 ± 1.5	AI	15	<0.05
	Men	4.2 ± 2.0		15	
B1 [mg]	Women	1.11 ± 0.42	EAR	0.9	>0.05
	Men	1.33 ± 0.52		1.1	
B2 [mg]	Women	1.31 ± 0.55	EAR	0.9	<0.05
	Men	1.54 ± 0.61		1.1	
B6 [mg]	Women	1.04 ± 0.29	EAR	1.1	<0.05
	Men	1.53 ± 0.41		1.3	
B12 [μg]	Women	3.02 ± 1.11	EAR	2.0	<0.05
	Men	4.21 ± 1.18		2.0	
Niacin [mg]	Women	18.7 ± 4.8	EAR	11	<0.05
	Men	22.1 ± 4.2		12	
C [mg]	Women	71.2 ± 32.6	EAR	60	<0.05
	Men	63.8 ± 27.9		75	
Folates [μg]	Women	225.4 ± 91.7	EAR	320	<0.05
	Men	242.1 ± 84.6		320	

Data are presented as mean ± standard deviation. Data distribution was tested using the Shapiro–Wilk test; all variables met normality assumptions, and comparisons between sexes were performed with the independent-samples Student’s *t*-test.

Conversely, the intake of several vitamins exceeded recommended dietary reference values. Mean consumption of vitamin A, vitamin B2, vitamin B12, niacin, and vitamin C was above the respective reference levels in both women and men. Vitamin E intake was adequate among women but remained below the recommended level in men.

Intake of vitamin B1 generally approximated recommended values in both sexes. In contrast, vitamin B6 intake was slightly below the estimated average requirement among women, whereas men exceeded the recommended intake level.

Overall, these results indicate that while the intake of most B-group vitamins and vitamin A is generally sufficient in IVDD patients, substantially inadequate intake persists for vitamin D and folates, representing the most prominent micronutrient inadequacies in this cohort.

The prevalence analysis demonstrated that vitamin inadequacies were common in both IVDD and LSS cohorts, although their distribution varied across specific vitamins (Table 8). The most pronounced inadequacy concerned vitamin D, with inadequate intake observed in the vast majority of participants in both disease groups and sexes. Similarly, inadequate folate intake was highly prevalent, affecting approximately two-thirds to three-quarters of patients regardless of diagnosis.

**Table 8:** Prevalence of inadequate vitamin intake in patients with IVDD and LSS, stratified by sex.

Group of patients	Vitamin	Women (%) [95% CI]	Men (%) [95% CI]
LSS	Vitamin A	28.7 (20.2–38.8)	17.2 (10.6–26.5)
	Vitamin E	44.8 (34.7–55.3)	68.8 (58.5–77.5)
	Vitamin D	96.5 (90.3–98.8)	92.5 (85.0–96.4)
	Vitamin B1	37.9 (28.3–48.5)	24.7 (17.0–34.4)
	Vitamin B2	18.4 (11.6–28.1)	12.9 (7.4–21.3)
	Vitamin B6	52.9 (42.4–63.1)	21.5 (14.4–31.0)
	Vitamin B12	9.2 (4.4–17.9)	4.3 (1.6–10.6)
	Niacin	6.9 (2.9–15.2)	3.2 (1.0–9.3)
	Vitamin C	33.3 (24.2–44.0)	48.4 (38.5–58.4)
	Folates	74.7 (64.7–82.6)	69.9 (59.7–78.4)
IVDD	Vitamin A	25.5 (17.9–34.9)	14.2 (8.8–22.1)
	Vitamin E	38.3 (28.9–48.7)	62.3 (52.6–71.1)
	Vitamin D	95.7 (89.6–98.3)	90.6 (83.4–94.8)
	Vitamin B1	32.9 (24.1–43.1)	21.7 (14.9–30.6)
	Vitamin B2	15.9 (9.6–25.2)	11.3 (6.5–18.9)
	Vitamin B6	49.0 (38.8–59.3)	18.9 (12.6–27.4)
	Vitamin B12	7.4 (3.5–14.8)	3.8 (1.4–9.8)
	Niacin	5.3 (2.1–12.8)	2.8 (0.9–8.2)
	Vitamin C	28.7 (20.6–38.6)	44.3 (34.9–54.1)
	Folates	71.3 (61.4–79.4)	66.0 (56.3–74.5)

Moderate levels of inadequacy were observed for vitamin E and vitamin B6. Inadequate vitamin E intake was particularly common among men in both cohorts, whereas vitamin B6 inadequacy was more frequent among women. Vitamin C inadequacy was also notable, especially among men.

In contrast, inadequate intake of vitamin A, vitamin B1, and vitamin B2 occurred at lower but still measurable frequencies. The prevalence of inadequate intake for vitamin B12 and niacin was relatively low in both patient populations.

Overall, these findings indicate that patients with degenerative spinal disorders frequently exhibit inadequate intake of vitamin D and folates, while inadequate intake of other vitamins occurs less consistently and show some sex-related variability.

Analysis of mineral intake in patients with intervertebral disc degeneration demonstrated several deviations from recommended dietary reference values (Table 9). Na intake substantially exceeded the adequate intake in both women and men, indicating excessive dietary Na consumption within the IVDD cohort.

**Table 9:** Mean daily mineral intake in patients with IVDD compared with dietary reference values.

Mineral component	Gender	Mean ± SD	Norm		*p*-value *t*-student’s test
			Type of standard	Quantity/day	
Na [mg/day]	Women	2831.40 ± 1475.30	AI	1,500	<0.05
	Men	3510.70 ± 1821.60		1,500	
K [mg/day]	Women	3720.80 ± 1584.60	AI	3,500	<0.05
	Men	3265.40 ± 1798.10		2,500	
Ca [mg/day]	Women	505.70 ± 120.30	EAR	800	<0.05
	Men	604.80 ± 231.90		1,000	
P [mg/day]	Women	1360.10 ± 455.70	EAR	580	>0.05
	Men	1475.30 ± 215.60		580	
Mg [mg/day]	Women	268.70 ± 54.60	EAR	265	>0.05
	Men	324.10 ± 87.90		350	
Zn [mg/day]	Women	9.10 ± 2.00	EAR	6.80	>0.05
	Men	10.90 ± 2.40		9.40	
Cu [mg/day]	Women	0.92 ± 0.23	EAR	0.70	>0.05
	Men	0.96 ± 0.20		0.70	
Mn [mg/day]	Women	3.90 ± 1.12	AI	1.80	>0.05
	Men	4.60 ± 1.05		2.30	
Fe [mg/day]	Women	9.00 ± 2.30	EAR	8	<0.05
	Men	12.90 ± 5.40		6	
I [μg/day]	Women	92.40 ± 34.80	EAR	95	>0.05
	Men	151.80 ± 42.10		95	

Na, sodium; K, potassium; Ca, calcium; P, phosphorus; Mg, magnesium; Zn, zinc; Cu, copper; Mn, manganese; Fe, iron; I, iodine.

Ca intake was markedly below the estimated average requirement in both sexes, representing one of the most pronounced mineral inadequacies observed in the study population. In contrast, potassium intake generally exceeded the recommended adequate intake in women and approached recommended levels in men.

P intake was well above the reference value in both sexes, whereas magnesium intake approximated the recommended levels but remained slightly lower among men relative to the reference standard. Intake of Zn, Cu, and Mn exceeded their respective dietary requirements in both sexes.

Fe intake was above the estimated average requirement in both women and men, particularly among men. Iodine intake was close to the recommended level in women and exceeded the reference value in men.

Overall, the mineral intake profile in IVDD patients was characterized by excessive Na and P consumption, adequate or elevated intake of several trace elements, and marked inadequacy of Ca intake.

### 3.3 Mineral intake and prevalence of inadequacy in IVDD and LSS patients

Evaluation of mineral intake among patients with lumbar spinal stenosis revealed several deviations from recommended dietary intake levels (Table 10). Na consumption markedly exceeded the adequate intake values in both women and men, indicating consistently elevated dietary sodium intake within this patient population.

**Table 10:** Mean daily mineral intake in patients with LSS compared with dietary reference values.

Mineral component	Gender	Mean ± SD	Norm		*p*-value *t*-student’s test
			Type of standard	Quantity/day	
Na [mg/day]	Women (*n* = 87)	2745.60 ± 1389.50	AI	1,500	<0.05
	Men (*n* = 93)	3365.80 ± 1695.20		1,500	
K [mg/day]	Women	3612.40 ± 1501.80	AI	3,500	>0.05
	Men	3150.70 ± 1710.90		2,500	
Ca [mg/day]	Women	488.30 ± 115.20	EAR	800	<0.05
	Men	585.40 ± 218.60		1,000	
P [mg/day]	Women	1312.80 ± 432.60	EAR	580	>0.05
	Men	1426.50 ± 208.40		580	
Mg [mg/day]	Women	262.30 ± 52.40	EAR	265	>0.05
	Men	316.90 ± 83.70		350	
Zn [mg/day]	Women	8.70 ± 2.10	EAR	6.80	>0.05
	Men	10.50 ± 2.30		9.40	
Cu [mg/day]	Women	0.88 ± 0.22	EAR	0.70	>0.05
	Men	0.93 ± 0.21		0.70	
Mn [mg/day]	Women	3.70 ± 1.10	AI	1.80	>0.05
	Men	4.30 ± 1.00		2.30	
Fe [mg/day]	Women	8.60 ± 2.40	EAR	8	>0.05
	Men	12.30 ± 5.20		6	
I [μg/day]	Women	88.20 ± 32.90	EAR	95	<0.05
	Men	146.10 ± 40.80		95	

Na, sodium; K, potassium; Ca, calcium; P, phosphorus; Mg, magnesium; Zn, zinc; Cu, copper; Mn, manganese; Fe, iron; I, iodine.

Ca intake was substantially lower than the estimated average requirement in both sexes, representing the most pronounced mineral inadequacy identified in the LSS cohort. In contrast, K intake was generally close to recommended values, slightly exceeding the adequate intake in women while remaining above the reference level in men.

P intake was considerably higher than the estimated requirement in both sexes. Mg intake approximated recommended levels but remained slightly below the reference value in men. Similarly, intake Zn, Cu, Mn exceeded the established dietary reference values in both women and men.

Fe intake met or slightly exceeded the estimated requirement, particularly among men. Conversely, I intake was slightly below the recommended level in women but exceeded the reference value in men.

Overall, the mineral intake profile in LSS patients was characterized by excessive sodium and phosphorus consumption, adequate or elevated intake of several trace elements, and pronounced inadequacy of calcium intake, with marginally insufficient iodine intake observed among women.

The prevalence analysis revealed that several minerals were frequently consumed in inadequate amounts in both IVDD and LSS cohorts (Table 11). The most prominent inadequacy concerned calcium intake, with inadequacy observed in the vast majority of participants in both disease groups and sexes. More than 88% of women and over 92% of men in the LSS group, as well as over 90% of women and 94% of men in the IVDD group, consumed calcium below the recommended level.

**Table 11:** Prevalence of inadequate mineral intake in patients with IVDD and LSS, stratified by sex.

Group of patients	Mineral component	Women (%) [95% CI]	Men (%) [95% CI]
LSS	Na	0.0 (0.0–4.1)	0.0 (0.0–3.9)
	K	46.0 (35.9–56.4)	38.7 (29.5–48.8)
	Ca	88.5 (80.3–93.5)	92.4 (85.3–96.3)
	P	0.0 (0.0–4.1)	0.0 (0.0–3.9)
	Mg	48.3 (38.1–58.7)	64.5 (54.4–73.5)
	Zn	18.4 (11.6–28.1)	21.5 (14.4–31.0)
	Cu	9.2 (4.4–17.9)	8.6 (4.3–16.4)
	Mn	0.0 (0.0–4.1)	0.0 (0.0–3.9)
	Fe	42.5 (32.5–53.2)	8.6 (4.3–16.4)
	I	53.0 (42.4–63.3)	18.3 (11.9–27.1)
IVDD	Na	0.0 (0.0–3.1)	0.0 (0.0–2.9)
	K	44.7 (35.0–54.8)	36.8 (27.9–46.7)
	Ca	90.4 (82.8–94.9)	94.3 (87.9–97.4)
	P	0.0 (0.0–3.1)	0.0 (0.0–2.9)
	Mg	46.8 (37.1–56.7)	61.3 (51.5–70.2)
	Zn	16.0 (9.8–24.9)	20.8 (13.9–29.9)
	Cu	8.5 (4.3–16.0)	7.5 (3.6–14.6)
	Mn	0.0 (0.0–3.1)	0.0 (0.0–2.9)
	Fe	38.3 (28.9–48.7)	7.5 (3.6–14.6)
	I	50.0 (40.4–59.6)	16.0 (9.8–24.9)

Na, sodium; K, potassium; Ca, calcium; P, phosphorus; Mg, magnesium; Zn, zinc; Cu, copper; Mn, manganese; Fe, iron; I, iodine.

Moderate levels of inadequacy were observed for Mg and K. Approximately half of the women and between one-third and two-thirds of men in both cohorts did not reach recommended magnesium intake levels. K inadequacy affected roughly 37–46% of patients across both diagnostic groups.

Inadequate Fe intake showed a clear sex-related pattern. Among women, inadequate intake was relatively common (approximately 38–43%), whereas it occurred much less frequently among men (about 7–9%). I inadequacy was also notable, particularly among women, affecting approximately half of female patients in both IVDD and LSS groups.

In contrast, inadequate intake of Na, P, and Mn was not observed in either cohort, indicating that intake of these minerals generally exceeded recommended dietary levels. Inadequate Zn and Cu intake occurred less frequently, affecting roughly 7–22% of patients.

**Figure 1:**
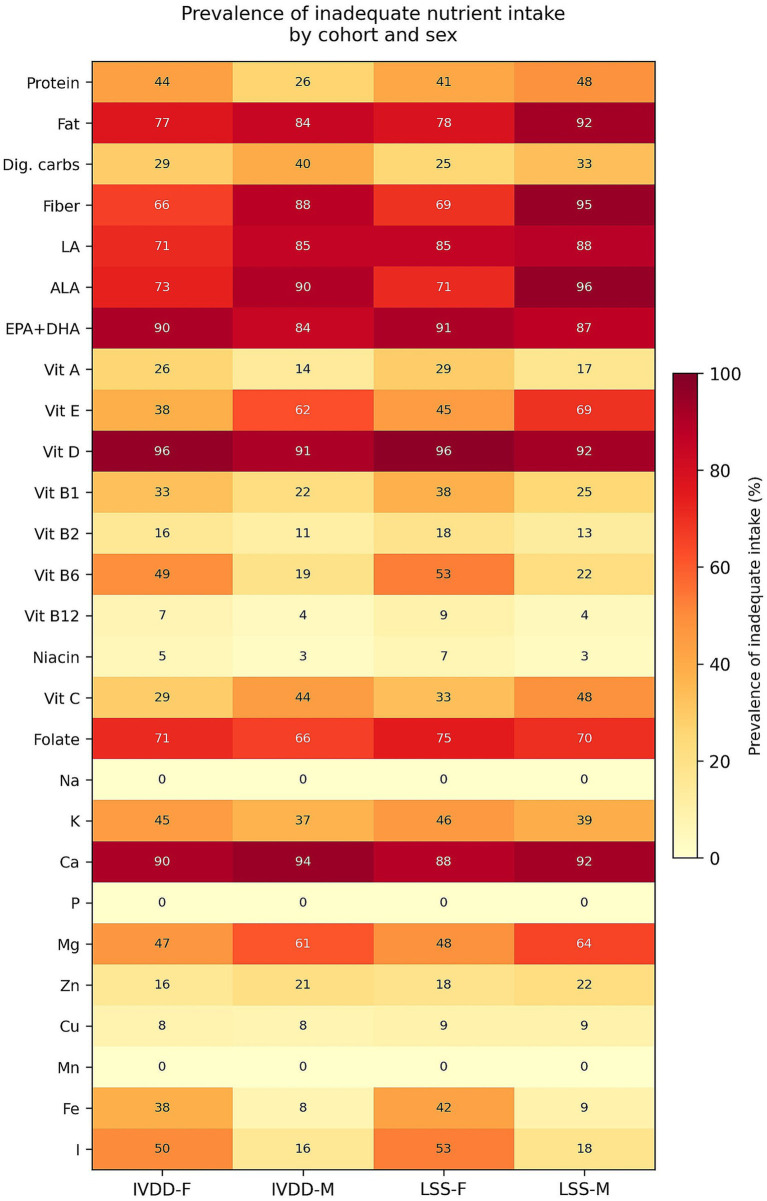
Prevalence of inadequate nutrient intake by cohort and sex. Heatmap of macronutrients, essential fatty acids, vitamins, and minerals; values correspond to Tables 5, 8, and 11.

**Figure 2:**
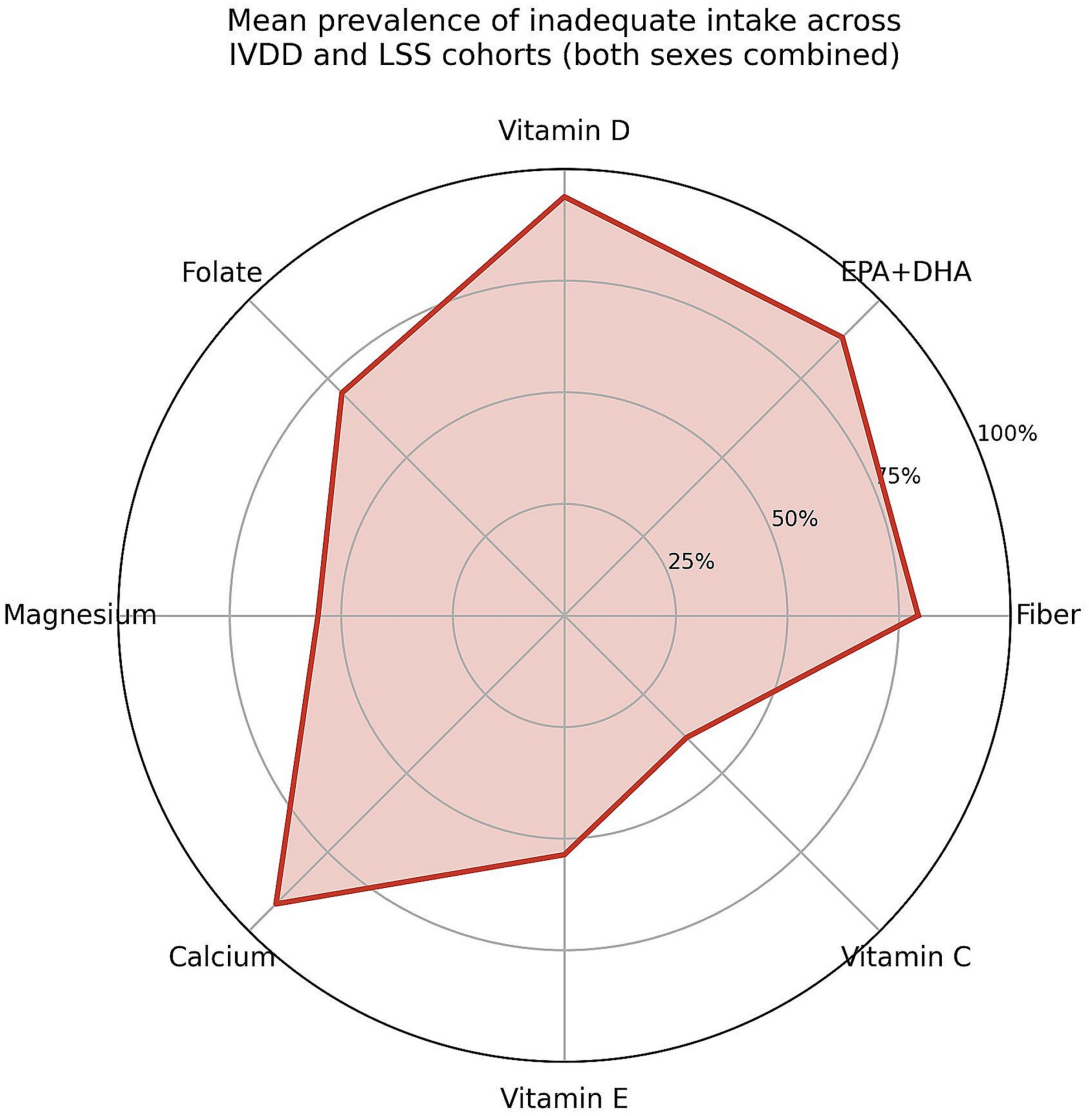
Radar summary of key nutrient inadequacies. Mean prevalence across IVDD and LSS cohorts (both sexes combined) for eight key nutrients.

Overall, the mineral intake profile in both spinal disorder cohorts was characterized by widespread calcium inadequacy, moderate inadequacy of magnesium and potassium, and sex-related differences in iron and iodine intake. The full prevalence pattern across all macronutrients, essential fatty acids, vitamins, and minerals described above (Tables 5, 8, 11) is summarized graphically in Figure 1, and the relative magnitude of inadequacy for the eight nutrients most consistently affected is illustrated in the radar plot in Figure 2.

### 3.4 Adjusted associations between nutrient inadequacy and clinical outcomes

To evaluate the relationship between nutrient intake and clinical/radiological severity while avoiding the interpretive and statistical limitations inherent to testing each nutrient in isolation, all seven nutrients most frequently affected by inadequate intake (EPA + DHA, fiber, folate, magnesium, vitamin D, calcium, and zinc) were entered simultaneously into multivariable regression models, together with age, sex, BMI, total energy intake, smoking status, diabetes, alcohol use, occupation type, and education level. Variance inflation factors for all predictors in these models ranged from 1.05 to 1.34, indicating no relevant multicollinearity among the candidate nutrients or between nutrients and covariates (Supplementary Table S2).

**Figure 3:**
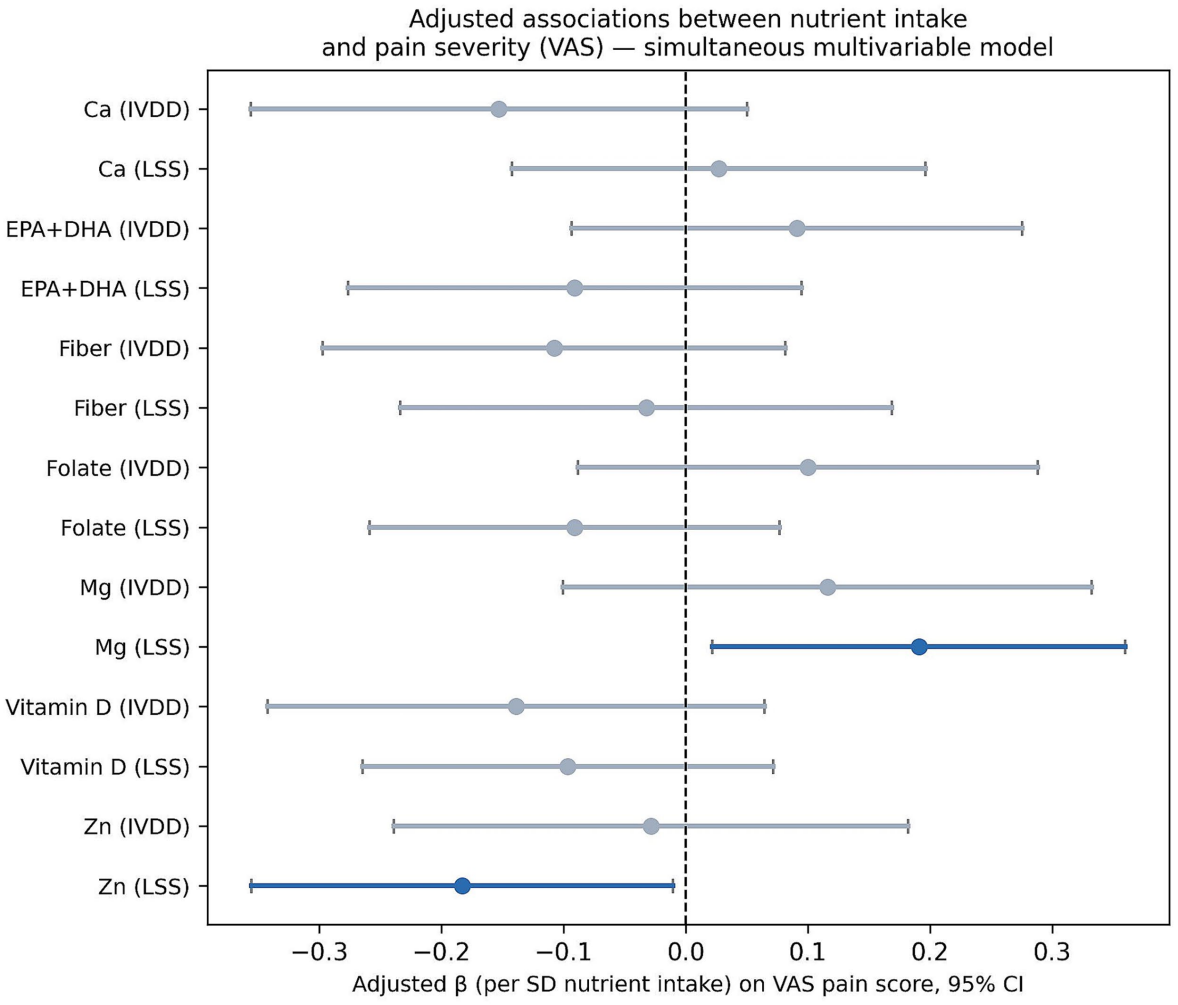
Adjusted associations between nutrient intake and pain severity. Forest plot of *β* coefficients (95% CI) from the simultaneous multivariable model (VAS, per SD nutrient intake, IVDD and LSS). Filled markers indicate nominal *p* < 0.05 (uncorrected); none significant after FDR. Corresponds to Table 12.

In the resulting simultaneous models, no nutrient retained a statistically significant independent association with VAS pain score after FDR correction, in either the IVDD cohort (model *R*^2^ = 0.64) or the LSS cohort (model *R*^2^ = 0.60; Table 12; Figure 3). Nominal, uncorrected associations (*p* < 0.05 before FDR correction) were observed for magnesium [*β* = 0.19 per SD, 95% CI (0.02, 0.36), *p* = 0.027] and zinc [*β* = −0.18 per SD, 95% CI (−0.36, −0.01), *p* = 0.038] with pain severity in the LSS cohort only; neither association survived correction for multiple comparisons (*q* = 0.13 for both).

**Table 12:** Adjusted associations between nutrient intake and pain severity/disc degeneration.

Outcome	Nutrient	Group	*β* (per SD)	95% CI	*p*	q (FDR)	*n*
VAS	EPA + DHA	IVDD	0.09	[−0.09, 0.28]	0.331	0.387	200
VAS	Fiber	IVDD	−0.11	[−0.30, 0.08]	0.263	0.387	200
VAS	Folate	IVDD	0.10	[−0.09, 0.29]	0.296	0.387	200
VAS	Mg	IVDD	0.12	[−0.10, 0.33]	0.291	0.387	200
VAS	Vitamin D	IVDD	−0.14	[−0.34, 0.06]	0.179	0.387	200
VAS	Ca	IVDD	−0.15	[−0.36, 0.05]	0.139	0.387	200
VAS	Zn	IVDD	−0.03	[−0.24, 0.18]	0.790	0.790	200
VAS	EPA + DHA	LSS	−0.09	[−0.28, 0.09]	0.335	0.469	180
VAS	Fiber	LSS	−0.03	[−0.23, 0.17]	0.752	0.753	180
VAS	Folate	LSS	−0.09	[−0.26, 0.08]	0.287	0.469	180
VAS	Mg	LSS	0.19	[0.02, 0.36]	0.027	0.132	180
VAS	Vitamin D	LSS	−0.10	[−0.26, 0.07]	0.259	0.469	180
VAS	Ca	LSS	0.03	[−0.14, 0.20]	0.753	0.753	180
VAS	Zn	LSS	−0.18	[−0.36, −0.01]	0.038	0.132	180
Pfirrmann grade	EPA + DHA	IVDD	−0.08	[−0.19, 0.04]	0.189	0.568	200
Pfirrmann grade	Fiber	IVDD	0.07	[−0.05, 0.19]	0.243	0.568	200
Pfirrmann grade	Folate	IVDD	0.05	[−0.07, 0.16]	0.432	0.605	200
Pfirrmann grade	Mg	IVDD	−0.01	[−0.14, 0.12]	0.888	0.888	200
Pfirrmann grade	Vitamin D	IVDD	0.05	[−0.07, 0.18]	0.391	0.605	200
Pfirrmann grade	Ca	IVDD	0.10	[−0.02, 0.23]	0.112	0.568	200
Pfirrmann grade	Zn	IVDD	0.02	[−0.10, 0.15]	0.706	0.824	200

Multivariable models in which all seven nutrients were entered simultaneously (per SD, continuous) together with age, sex, BMI, energy intake, smoking, diabetes, alcohol use, occupation type, and education, in IVDD and LSS cohorts. No association remained significant after FDR correction (*q* < 0.05). IVDD, intervertebral disc degeneration; LSS, lumbar spinal stenosis; VAS, visual analog scale; BMI, body mass index; SD, standard deviation; FDR, false discovery rate; EPA, eicosapentaenoic acid; DHA, docosahexaenoic acid; Mg, magnesium; Ca, calcium; Zn, zinc.

For structural disc degeneration in the IVDD cohort, the overall multivariable model was not statistically significant (*R*^2^ = 0.07, F-test *p* = 0.69), and none of the seven nutrients showed a significant association with Pfirrmann grade, either before or after FDR correction (Table 12).

In the LSS cohort, the same panel of nutrients was additionally examined in relation to radiological markers of stenosis severity available from preoperative MRI, including Schizas grade, ligamentum flavum thickness, anteroposterior canal diameter, dural sac cross-sectional area, and grades of central, facet, foraminal, and lateral recess stenosis (Table 13). As with the clinical pain outcome, no nutrient-outcome association remained significant after FDR correction. Nominal associations were observed for magnesium and zinc with ligamentum flavum thickness (*p* = 0.048 and *p* = 0.044, respectively) and for folate with dural sac area (*p* = 0.009); all corresponded to *q* > 0.05.

**Table 13:** Adjusted associations between nutrient intake and LSS radiological severity.

Radiological outcome	Nutrient	β (per SD)	95% CI	*p*	q (FDR)	*n*
Schizas grade	EPA + DHA	0.04	[−0.05, 0.13]	0.407	0.771	180
Schizas grade	Fiber	−0.02	[−0.11, 0.08]	0.749	0.930	180
Schizas grade	Folate	−0.05	[−0.13, 0.03]	0.201	0.771	180
Schizas grade	Mg	0.03	[−0.05, 0.11]	0.440	0.771	180
Schizas grade	Vitamin D	0.01	[−0.07, 0.09]	0.874	0.930	180
Schizas grade	Ca	−0.00	[−0.08, 0.08]	0.930	0.930	180
Schizas grade	Zn	0.03	[−0.05, 0.12]	0.440	0.771	180
LF thickness (mm)	EPA + DHA	−0.07	[−0.17, 0.03]	0.154	0.269	180
LF thickness (mm)	Fiber	−0.07	[−0.18, 0.04]	0.207	0.290	180
LF thickness (mm)	Folate	0.01	[−0.09, 0.10]	0.894	0.894	180
LF thickness (mm)	Mg	0.09	[0.00, 0.19]	0.048	0.168	180
LF thickness (mm)	Vitamin D	−0.05	[−0.14, 0.04]	0.264	0.308	180
LF thickness (mm)	Ca	0.07	[−0.02, 0.16]	0.120	0.269	180
LF thickness (mm)	Zn	−0.10	[−0.19, −0.00]	0.044	0.168	180
AP canal diameter (mm)	EPA + DHA	0.06	[−0.15, 0.27]	0.578	0.949	180
AP canal diameter (mm)	Fiber	0.02	[−0.21, 0.24]	0.885	0.949	180
AP canal diameter (mm)	Folate	0.11	[−0.08, 0.30]	0.252	0.882	180
AP canal diameter (mm)	Mg	−0.13	[−0.32, 0.06]	0.189	0.882	180
AP canal diameter (mm)	Vitamin D	0.02	[−0.17, 0.21]	0.851	0.949	180
AP canal diameter (mm)	Ca	−0.01	[−0.20, 0.18]	0.949	0.949	180
AP canal diameter (mm)	Zn	0.08	[−0.11, 0.28]	0.406	0.948	180
Dural sac area (mm^2^)	EPA + DHA	0.34	[−1.86, 2.55]	0.758	0.811	180
Dural sac area (mm^2^)	Fiber	0.29	[−2.10, 2.68]	0.811	0.811	180
Dural sac area (mm^2^)	Folate	2.61	[0.65, 4.57]	0.009	0.065	180
Dural sac area (mm^2^)	Mg	−1.31	[−3.33, 0.71]	0.204	0.588	180
Dural sac area (mm^2^)	Vitamin D	−0.33	[−2.33, 1.67]	0.742	0.811	180
Dural sac area (mm^2^)	Ca	−0.48	[−2.49, 1.53]	0.638	0.811	180
Dural sac area (mm^2^)	Zn	1.20	[−0.86, 3.26]	0.252	0.588	180
Central stenosis grade	EPA + DHA	0.04	[−0.05, 0.13]	0.407	0.771	180
Central stenosis grade	Fiber	−0.02	[−0.11, 0.08]	0.749	0.930	180
Central stenosis grade	Folate	−0.05	[−0.13, 0.03]	0.201	0.771	180
Central stenosis grade	Mg	0.03	[−0.05, 0.11]	0.440	0.771	180
Central stenosis grade	Vitamin D	0.01	[−0.07, 0.09]	0.874	0.930	180
Central stenosis grade	Ca	−0.00	[−0.08, 0.08]	0.930	0.930	180
Central stenosis grade	Zn	0.03	[−0.05, 0.12]	0.440	0.771	180
Facet arthrosis grade	EPA + DHA	0.03	[−0.07, 0.14]	0.544	0.760	180
Facet arthrosis grade	Fiber	−0.08	[−0.19, 0.03]	0.154	0.506	180
Facet arthrosis grade	Folate	−0.06	[−0.15, 0.04]	0.217	0.506	180
Facet arthrosis grade	Mg	0.08	[−0.02, 0.17]	0.105	0.506	180
Facet arthrosis grade	Vitamin D	−0.01	[−0.11, 0.08]	0.760	0.760	180
Facet arthrosis grade	Ca	−0.02	[−0.12, 0.08]	0.677	0.760	180
Facet arthrosis grade	Zn	−0.04	[−0.14, 0.05]	0.367	0.643	180
Foraminal stenosis grade	EPA + DHA	−0.05	[−0.15, 0.05]	0.344	0.647	180
Foraminal stenosis grade	Fiber	−0.06	[−0.17, 0.04]	0.244	0.647	180
Foraminal stenosis grade	Folate	−0.03	[−0.12, 0.06]	0.485	0.679	180
Foraminal stenosis grade	Mg	0.04	[−0.05, 0.13]	0.370	0.647	180
Foraminal stenosis grade	Vitamin D	0.00	[−0.09, 0.09]	0.992	0.992	180
Foraminal stenosis grade	Ca	0.01	[−0.08, 0.10]	0.775	0.905	180
Foraminal stenosis grade	Zn	−0.09	[−0.18, 0.00]	0.055	0.388	180
Lateral recess stenosis grade	EPA + DHA	0.03	[−0.09, 0.14]	0.635	0.801	180
Lateral recess stenosis grade	Fiber	0.06	[−0.06, 0.18]	0.359	0.801	180
Lateral recess stenosis grade	Folate	−0.09	[−0.19, 0.01]	0.076	0.532	180
Lateral recess stenosis grade	Mg	0.02	[−0.08, 0.12]	0.687	0.801	180
Lateral recess stenosis grade	Vitamin D	0.01	[−0.10, 0.11]	0.904	0.904	180
Lateral recess stenosis grade	Ca	0.03	[−0.07, 0.13]	0.533	0.801	180
Lateral recess stenosis grade	Zn	0.02	[−0.08, 0.13]	0.642	0.801	180

Multivariable models (per SD, continuous) adjusted as in Table 12, LSS cohort (*n* = 180). Schizas grade coded A = 1 to D = 4. No association remained significant after FDR correction. LSS, lumbar spinal stenosis; SD, standard deviation; EPA, eicosapentaenoic acid; DHA, docosahexaenoic acid; Mg, magnesium; Ca, calcium; Zn, zinc; LF, ligamentum flavum; AP, anteroposterior.

Overall, despite the high prevalence of inadequate intake of vitamin D, fiber, calcium, and EPA + DHA described above, none of these nutrients showed a statistically robust, independent association with pain severity, disc degeneration, or radiological stenosis severity once all nutrients were modeled simultaneously and adjusted for a comprehensive set of demographic, anthropometric, and lifestyle covariates. The confidence intervals around most estimates were compatible with both the absence of an effect and with a moderate effect in either direction (Table 12), consistent with limited statistical power for detecting independent, nutrient-specific effects at the present sample size once a broader covariate panel is included.

Additional covariate analyses are presented in the Supplementary Tables S2, S3. These models evaluated the influence of demographic and clinical factors on the associations between nutrient adequacy and clinical outcomes. In both IVDD and LSS cohorts, baseline pain severity was the strongest predictor of follow-up VAS scores across all nutrient models (*β* ≈ 0.91–0.97, *p* < 0.001). Body mass index and age showed modest positive associations with pain severity, particularly in the fiber models, whereas sex demonstrated a small effect only in the vitamin D models. Baseline energy intake did not show a significant association with pain outcomes. In the IVDD cohort, analyses of Pfirrmann grade indicated that age was consistently associated with more advanced disc degeneration across all models (*p* < 0.001), while BMI showed weaker but significant effects in selected models (e.g., fiber and calcium). Baseline Pfirrmann grade strongly predicted follow-up degeneration severity (*β* ≈ 0.89–0.90, *p* < 0.001). Overall, these supplementary analyses confirm that the observed associations between nutrient inadequacy and clinical outcomes were independent of major demographic and metabolic covariates.

## 4 Discussion

**Figure 4:**
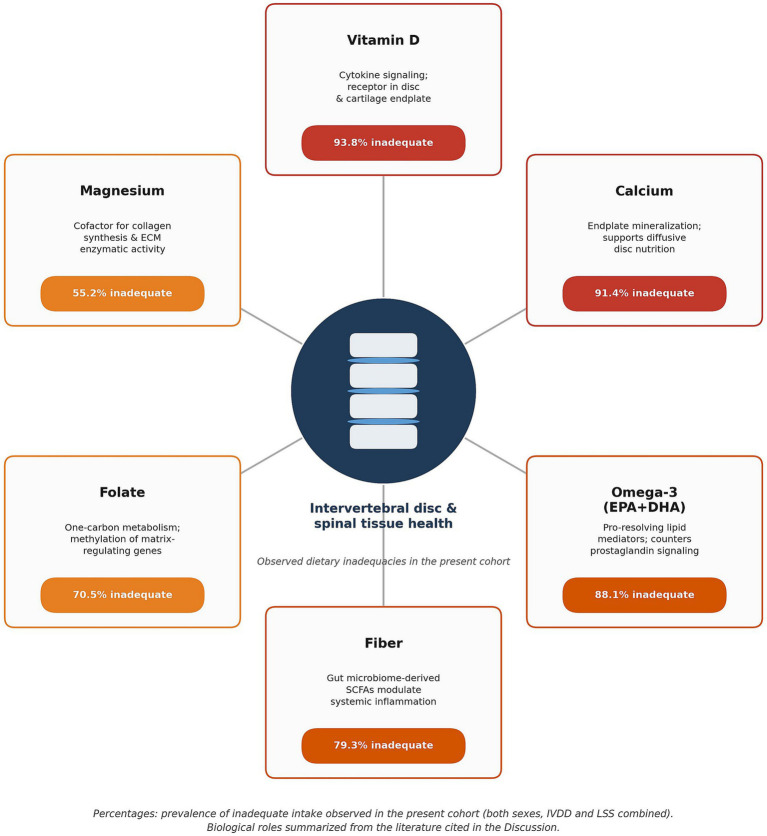
Key nutrients in intervertebral disc and spinal tissue physiology. Schematic summary of proposed biological roles (Vitamin D, Calcium, Magnesium, Fiber, Omega-3, Folate) and their prevalence of inadequate intake in this cohort.

The present study provides a comprehensive assessment of dietary patterns and nutrient intake in patients with IVDD and LSS, revealing a consistent profile of nutritional imbalance characterized by insufficient intake of several essential nutrients and excessive consumption of selected dietary components. These findings suggest that metabolic and nutritional disturbances may represent an underrecognized factor associated with the development and progression of degenerative spinal disorders. The proposed biological roles of the six nutrients discussed below in relation to intervertebral disc and spinal connective tissue physiology, together with their prevalence of inadequate intake in the present cohort, are summarized schematically in Figure 4.

One of the most consistent observations in the present analysis was the reduced total daily energy intake relative to estimated physiological requirements, accompanied by an unfavorable macronutrient distribution characterized by relatively high fat intake and reduced carbohydrate contribution. Such dietary patterns may contribute to metabolic dysregulation and chronic low-grade inflammation, which are increasingly recognized as important contributors to degenerative musculoskeletal diseases (38–40). Persistent inflammatory activation may influence ECM turnover in spinal tissues through stimulation of catabolic signaling pathways and increased expression of matrix-degrading enzymes (6). Consequently, nutritional imbalance may indirectly accelerate degenerative processes by promoting inflammatory and oxidative mechanisms that impair tissue repair and structural integrity (41).

A particularly important finding was the widespread insufficiency of long-chain polyunsaturated fatty acids, especially EPA and DHA. These fatty acids are critical regulators of inflammatory responses and serve as precursors for lipid mediators that modulate cytokine production and immune signaling (42–44). Reduced intake of EPA and DHA may therefore favor a pro-inflammatory biochemical environment characterized by increased production of cytokines such as IL-1β and TNF-*α*, both of which are known to participate in disc degeneration and ligament hypertrophy (45, 46). Experimental studies indicate that omega-3 fatty acids may attenuate inflammatory signaling cascades and reduce catabolic activity within connective tissues, suggesting that insufficient intake of these nutrients could exacerbate degenerative changes within spinal structures (47, 48), although no independent association between EPA + DHA intake and pain severity or disc degeneration was demonstrated in our adjusted analyses.

Another important nutritional inadequacy identified in the present cohort was insufficient intake of dietary fiber, particularly among male participants. Dietary fiber plays a key role in metabolic regulation, glucose homeostasis, and maintenance of gut microbiota composition (49, 50). Increasing evidence indicates that gut microbiota-derived metabolites such as short-chain fatty acids exert anti-inflammatory effects and modulate systemic immune responses (51, 52). Therefore, insufficient fiber intake may contribute to metabolic imbalance and systemic inflammation (53), which may indirectly influence degenerative processes affecting spinal tissues, although no independent association between fiber intake and pain severity or disc degeneration was demonstrated in our adjusted analyses.

The analysis of vitamin intake revealed particularly pronounced inadequacy of vitamin D intake in both IVDD and LSS groups. Vitamin D plays a fundamental role in musculoskeletal physiology through its involvement in Ca metabolism, bone remodeling, and neuromuscular regulation. In addition to its skeletal functions, vitamin D exerts important immunomodulatory and anti-inflammatory effects. Deficiency of this vitamin has been associated with increased musculoskeletal pain, impaired muscle strength, and structural degeneration within spinal tissues (54–56). Moreover, vitamin D influences gene expression involved in inflammatory signaling and ECM metabolism, suggesting that chronic deficiency may contribute to progressive degeneration and pain sensitization (57, 58).

These mechanistic pathways provide biological plausibility for a link between vitamin D status and spinal pain and degeneration. In the present cohort, however, inadequate vitamin D intake was not independently associated with VAS pain score or Pfirrmann grade once modeled simultaneously with other nutrients and adjusted for the full covariate panel, indicating that any such relationship, if present, was not detectable with the statistical power available in this cross-sectional sample.

Another highly prevalent inadequacy identified in this population was insufficient folate intake. Folate plays a central role in one-carbon metabolism, which regulates DNA synthesis, repair mechanisms, and epigenetic processes including methylation (59). Inadequate folate intake may impair cellular regeneration and promote oxidative stress within connective tissues. Additionally, folate deficiency may lead to elevated homocysteine levels, which can adversely affect endothelial function and microvascular circulation (60). Impaired microcirculation has been proposed as a contributing factor in disc degeneration, suggesting that chronic folate insufficiency may indirectly influence structural deterioration of spinal tissues (61–63). Consistent with this uncertainty, inadequate folate intake was not independently associated with pain severity or disc degeneration in the present cohort, either before or after adjustment for the full covariate panel.

The mineral intake profile also revealed several noteworthy imbalances. In particular, Na intake frequently exceeded recommended dietary levels, whereas Ca intake remained markedly insufficient in both sexes. Excessive Na intake has been associated with vascular dysfunction, oxidative stress, and inflammatory activation, which may indirectly contribute to degenerative tissue changes (64, 65). In contrast, inadequate Ca intake may impair bone metabolism and compromise skeletal stability, potentially influencing vertebral structure and biomechanical properties of the spinal column (66). Another notable observation was the elevated intake of P, which often occurred simultaneously with insufficient Ca intake. Disruption of the Ca/P balance may affect mineral metabolism and bone remodeling processes, potentially contributing to structural alterations within vertebral tissues (22, 23, 67). Such imbalances may influence endocrine regulatory mechanisms governing mineral homeostasis and may therefore affect skeletal integrity and spinal stability (23, 68). In addition, variability in intake of trace elements such as Mg, Fe, and Zn was observed across the analyzed cohorts. These elements participate in numerous biological processes relevant to musculoskeletal health, including enzymatic antioxidant defense, mitochondrial metabolism, and collagen synthesis (69–75). Disturbances in these micronutrients may therefore contribute to oxidative stress and impaired connective tissue regeneration within degenerative spinal environments (69–75). However, as with the other nutrients discussed above, no independent association between calcium intake and pain severity or disc degeneration was demonstrated in our adjusted analyses, and these mineral-related mechanisms should likewise be regarded as biologically plausible rather than confirmed in the present cohort.

Sex-related differences in nutrient intake were also evident, with male participants demonstrating higher prevalence of inadequate intake for several key dietary components. These disparities likely reflect differences in dietary behavior and food choices between sexes (76). Greater consumption of processed foods and lower intake of plant-based products among men may contribute to reduced intake of fiber and essential fatty acids, thereby amplifying metabolic and inflammatory disturbances associated with degenerative spinal diseases (77–79).

Collectively, the findings of the present study indicate that nutritional imbalance may represent an important systemic component of degenerative spinal disorders. Although current treatment strategies for IVDD and LSS primarily focus on pharmacological management, physiotherapy, and surgical interventions, dietary factors are rarely considered in clinical management algorithms. However, given the strong biological links between nutrient status, inflammation, oxidative stress, and ECM remodeling, nutritional optimization may represent a valuable complementary strategy for improving metabolic health and potentially mitigating disease progression (80–82). Future studies should explore the potential effects of targeted nutritional interventions on clinical outcomes in patients with degenerative spinal disorders. Longitudinal investigations integrating dietary assessment with biochemical markers of nutrient status, inflammatory mediators, and molecular indicators of tissue degeneration may provide deeper insight into the complex interactions between nutrition and spinal pathology. Such approaches may ultimately support the development of multidisciplinary therapeutic strategies that incorporate nutritional assessment as a routine component of patient care.

This study was designed to test a biologically well-motivated hypothesis, grounded in established mechanistic evidence linking nutrient status to connective tissue and inflammatory pathways relevant to spinal degeneration; its inability to confirm this hypothesis under rigorous, simultaneous multivariable adjustment should be interpreted in light of the following limitations, including the statistical power constraints discussed below.

Several limitations should be acknowledged. First, the cross-sectional design does not allow causal relationships between nutrient intake and disease severity to be established, and reverse causation cannot be excluded: patients with more severe pain may adopt less favorable dietary patterns, reduce physical activity, or experience appetite changes secondary to their condition, rather than nutrient inadequacy driving disease severity. Second, only dietary intake was assessed; no serum biomarkers of nutritional status (e.g., 25-hydroxyvitamin D, serum folate, ionized calcium) were measured, and dietary intake does not necessarily equate to biochemical deficiency. For this reason, we have used the term “inadequate intake” rather than “deficiency” throughout this manuscript. Third, this cohort comprised exclusively surgically treated patients with advanced IVDD or LSS; the findings cannot be generalized to conservatively managed patients, the general low back pain population, or asymptomatic individuals, and likely reflect a selected population with more severe structural disease. Fourth, although the multivariable models adjusted for age, sex, BMI, total energy intake, smoking, diabetes, alcohol use, occupation type, and education, information on physical activity level, medication use (including NSAIDs and corticosteroids), comorbid inflammatory disease, and depression/anxiety was not available and could not be included; residual confounding from these unmeasured factors cannot be excluded. Fifth, dietary intake was assessed using self-reported 24-h recalls, which may be affected by recall bias and underreporting; although three repeated recalls were used to improve reliability, no additional external validation of the recall instrument was performed within this cohort. Sixth, and most importantly for interpretation of the present results, when all seven nutrients were modeled simultaneously rather than individually, none retained a statistically significant independent association with pain severity, disc degeneration, or radiological stenosis severity after correction for multiple comparisons (Tables 12, 13). The 95% confidence intervals for most estimates were wide and compatible with both a null effect and a moderate effect in either direction, indicating that the present sample size, once a broad covariate panel is included, may be underpowered to detect independent, nutrient-specific effects; absence of a statistically significant association in this analysis therefore cannot be equated with absence of any true association. The nominal (uncorrected) associations observed for magnesium and zinc with pain severity and ligamentum flavum thickness in the LSS cohort should be regarded as hypothesis-generating rather than confirmatory. Finally, the clinical significance of the originally observed univariate differences in nutrient intake warrants comment: for context, commonly cited minimal clinically important difference (MCID) thresholds for the VAS in chronic low back pain range from approximately 1.5 to 2.0 points, against which even the largest adjusted point estimates in the present analysis (all <0.2 points per SD of nutrient intake) would not, on their own, be considered clinically meaningful. Future prospective studies incorporating serum biomarkers, larger and more diverse samples, and a more complete confounder panel are needed to clarify whether an independent relationship between nutrient status and clinical or radiological severity of degenerative spinal disease exists.

Overall, the present findings demonstrate that substantial nutritional inadequacies are common among patients with IVDD and LSS and may contribute to metabolic and inflammatory conditions that influence spinal degeneration. These results highlight the potential value of integrating nutritional assessment and dietary counseling into the multidisciplinary management of degenerative spinal disorders (83).

## 5 Conclusion

This study was conceived and adequately powered to test a biologically well-motivated hypothesis, grounded in established mechanistic evidence linking nutrient status to connective tissue and inflammatory pathways relevant to spinal degeneration. That this hypothesis was not confirmed under rigorous, simultaneous multivariable adjustment reflects the statistical stringency applied rather than a deficiency in study design and underscores the importance of transparently reporting null findings in this emerging field.

Patients with IVDD and LSS demonstrate widespread inadequate intake of EPA + DHA, dietary fiber, vitamin D, folate, and calcium, together with excessive intake of Na and P. When these nutrients were evaluated simultaneously in multivariable models adjusted for a comprehensive panel of demographic, anthropometric, and lifestyle covariates, none showed a statistically significant independent association with pain severity, disc degeneration, or radiological stenosis severity after correction for multiple comparisons; nominal, uncorrected signals for magnesium and zinc in the LSS cohort require confirmation in future studies.

Mechanistically, inadequate intake of anti-inflammatory fatty acids and dietary fiber may contribute to chronic low-grade inflammation and the activation of inflammatory signaling pathways, such as nuclear factor kappa B (NF-κB) and mitogen-activated protein kinase (MAPK), which regulate cytokine production and extracellular matrix (ECM) degradation in spinal tissues. Concurrent inadequate intake of vitamin D and Ca, combined with excessive Na and P intake, may theoretically disrupt mineral homeostasis and musculoskeletal integrity; however, these mechanistic pathways remain speculative in the context of the present findings, since no independent statistical association between these nutrients and pain or degeneration severity was confirmed in this cohort after adjustment.

Nonetheless, the sheer prevalence of dietary inadequacy in this surgically treated population is, in itself, a clinically actionable finding: it identifies a modifiable, low-cost target for multidisciplinary management, independent of whether a direct causal relationship with pain or degeneration severity is ultimately confirmed. Integrating consideration of nutritional screening and dietary guidance into the care of patients with degenerative spinal disease may therefore be a reasonable adjunct strategy on general health grounds, while its specific effect on spinal pain and degeneration remains to be established. Future prospective studies incorporating serum biomarkers of nutritional status, larger and more heterogeneous samples (including conservatively managed and non-surgical patients), and a broader confounder panel are needed to determine whether a genuine, independent relationship between nutrient status and the clinical or radiological severity of IVDD and LSS exists.

## Data Availability

The original contributions presented in the study are included in the article/Supplementary material, further inquiries can be directed to the corresponding author.
