# Supplementary material for: Dietary inadequacies and their association with pain severity and disc degeneration in patients surgically treated for degenerative lumbosacral spine disorders
Source: Front Nutr. 2026 Jul 30;13:1910742. doi: 10.3389/fnut.2026.1910742 (PMC13469442; doi:10.3389/fnut.2026.1910742)
Supplement: Supplementary file 1 [file Table_1.DOCX]

Supplementary Material

Table S1. Categorization of food consumption frequency used for dietary pattern analysis in the study population.

| Food Group No. | Food Group Name | Example Products | Group I (Infrequent Consumption) | Group II (Frequent Consumption) |
| --- | --- | --- | --- | --- |
| 1 | White bread | white wheat bread | ≤1 time/week | ≥2–3 times/week |
| 2 | Wholemeal bread | rye bread | ≤1 time/week | ≥2–3 times/week |
| 3 | Sweets | sweet rolls, cakes, chocolate | ≤1 time/week | ≥2–3 times/week |
| 4 | Flour-based dishes | dumplings, pasta, pancakes | ≤1 time/week | ≥2–3 times/week |
| 5 | Groats/rice/cereals | barley groats, rice, oatmeal | ≤1 time/week | ≥2–3 times/week |
| 6 | Milk and dairy drinks | milk, yoghurt, kefir | ≤1 time/week | ≥2–3 times/week |
| 7 | Cottage cheese | quark | ≤1 time/week | ≥2–3 times/week |
| 8 | Ripened/processed cheese | cheddar, processed cheese | ≤1 time/week | ≥2–3 times/week |
| 9 | Meat | beef, pork | ≤1 time/week | ≥2–3 times/week |
| 10 | Poultry | chicken, turkey | ≤1 time/week | ≥2–3 times/week |
| 11 | Offal | liver, kidneys | ≤1 time/week | ≥2–3 times/week |
| 12 | Sausages | regular sausages | ≤1 time/week | ≥2–3 times/week |
| 13 | High-quality cold cuts | ham, smoked meats | ≤1 time/week | ≥2–3 times/week |
| 14 | Meat products | minced meat products | ≤1 time/week | ≥2–3 times/week |
| 15 | Bacon/lard | fatback, lard | ≤1 time/week | ≥2–3 times/week |
| 16 | Canned meat | all types | Excluded | Excluded |
| 17 | Canned fish | tuna, sardines | ≥1 time/week | ≥2–3 times/week |
| 18 | Fresh fish | salmon, cod | ≥1 time/week | ≥2–3 times/week |
| 19 | Eggs | hen eggs | ≤1 time/week | ≥2–3 times/week |
| 20 | Butter | butter | ≤1 time/week | ≥2–3 times/week |
| 21 | Margarine | soft margarine | ≤1 time/week | ≥2–3 times/week |
| 22 | Vegetable oils | sunflower oil, rapeseed oil | ≤1 time/week | ≥2–3 times/week |
| 23 | Olive oil | olive oil | ≤1 time/week | ≥2–3 times/week |
| 24 | Potatoes | boiled potatoes | ≤1 time/week | ≥2–3 times/week |
| 25 | Raw vegetables | cucumber, tomato | ≤1 time/week | ≥2–3 times/week |
| 26 | Cooked vegetables | boiled carrots, cabbage | ≤1 time/week | ≥2–3 times/week |
| 27 | Fruit | apples, berries | ≤1 time/week | ≥2–3 times/week |
| 28 | Fruit juices/drinks | orange juice, apple juice | ≤1 time/week | ≥2–3 times/week |
| 29 | Jam | all types | ≤1 time/week | ≥2–3 times/week |
| 30 | Honey | honey | ≤1 time/week | ≥2–3 times/week |
| 31 | Sugar in beverages | sugar in tea or coffee | ≤1 time/week | ≥2–3 times/week |
| 32 | Legume products | beans, lentils | ≤1 time/week | ≥2–3 times/week |
| 33 | Beer | beer | ≤1 time/week | ≥2–3 times/week |
| 34 | Wine | wine | Excluded | Excluded |
| 35 | Vodka | vodka | Excluded | Excluded |
| 36 | Tea | black tea | ≤1 time/week | ≥2–3 times/week |
| 37 | Coffee | coffee | ≤1 time/week | ≥2–3 times/week |

**Table S2.** Variance inflation factors (VIF) for all predictors in the three simultaneous multivariable models reported in Table 12 of the main text (IVDD – VAS, LSS – VAS, IVDD – Pfirrmann grade).

| Model | Predictor | VIF |
| --- | --- | --- |
| IVDD – VAS | EPA+DHA | 1.11 |
| IVDD – VAS | Fiber | 1.13 |
| IVDD – VAS | Folate | 1.07 |
| IVDD – VAS | Magnesium | 1.31 |
| IVDD – VAS | Vitamin D | 1.20 |
| IVDD – VAS | Calcium | 1.15 |
| IVDD – VAS | Zinc | 1.25 |
| IVDD – VAS | Age | 1.09 |
| IVDD – VAS | Sex (male) | 2.64 |
| IVDD – VAS | BMI | 1.05 |
| IVDD – VAS | Energy intake | 2.15 |
| IVDD – VAS | Smoking | 1.06 |
| IVDD – VAS | Diabetes | 1.07 |
| IVDD – VAS | Alcohol use | 1.16 |
| IVDD – VAS | Occupation (physical) | 1.06 |
| IVDD – VAS | Education (secondary) | 2.24 |
| IVDD – VAS | Education (higher) | 2.22 |
| LSS – VAS | EPA+DHA | 1.12 |
| LSS – VAS | Fiber | 1.13 |
| LSS – VAS | Folate | 1.06 |
| LSS – VAS | Magnesium | 1.16 |
| LSS – VAS | Vitamin D | 1.13 |
| LSS – VAS | Calcium | 1.15 |
| LSS – VAS | Zinc | 1.20 |
| LSS – VAS | Age | 1.15 |
| LSS – VAS | Sex (male) | 2.47 |
| LSS – VAS | BMI | 1.08 |
| LSS – VAS | Energy intake | 2.02 |
| LSS – VAS | Smoking | 1.11 |
| LSS – VAS | Diabetes | 1.04 |
| LSS – VAS | Alcohol use | 1.17 |
| LSS – VAS | Occupation (physical) | 1.04 |
| LSS – VAS | Education (secondary) | 1.70 |
| LSS – VAS | Education (higher) | 1.71 |
| IVDD – Pfirrmann grade | EPA+DHA | 1.11 |
| IVDD – Pfirrmann grade | Fiber | 1.13 |
| IVDD – Pfirrmann grade | Folate | 1.07 |
| IVDD – Pfirrmann grade | Magnesium | 1.31 |
| IVDD – Pfirrmann grade | Vitamin D | 1.20 |
| IVDD – Pfirrmann grade | Calcium | 1.15 |
| IVDD – Pfirrmann grade | Zinc | 1.25 |
| IVDD – Pfirrmann grade | Age | 1.09 |
| IVDD – Pfirrmann grade | Sex (male) | 2.64 |
| IVDD – Pfirrmann grade | BMI | 1.05 |
| IVDD – Pfirrmann grade | Energy intake | 2.15 |
| IVDD – Pfirrmann grade | Smoking | 1.06 |
| IVDD – Pfirrmann grade | Diabetes | 1.07 |
| IVDD – Pfirrmann grade | Alcohol use | 1.16 |
| IVDD – Pfirrmann grade | Occupation (physical) | 1.06 |
| IVDD – Pfirrmann grade | Education (secondary) | 2.24 |
| IVDD – Pfirrmann grade | Education (higher) | 2.22 |

*BMI, body mass index; VIF, variance inflation factor; IVDD, intervertebral disc degeneration; LSS, lumbar spinal stenosis.*

**Table S3.** Pearson correlation matrix among the seven nutrient intake variables (continuous, standardized) used in the simultaneous multivariable models. All pairwise correlations are below 0.15 in absolute value, confirming that the nutrients analyzed are not meaningfully collinear.

|  | EPA+DHA | Fiber | Folate | Magnesium | Vitamin D | Calcium | Zinc |
| --- | --- | --- | --- | --- | --- | --- | --- |
| EPA+DHA | 1.00 | -0.02 | -0.01 | 0.03 | 0.08 | 0.06 | 0.08 |
| Fiber | -0.02 | 1.00 | -0.03 | -0.07 | -0.08 | 0.02 | -0.12 |
| Folate | -0.01 | -0.03 | 1.00 | -0.07 | 0.08 | 0.02 | 0.02 |
| Magnesium | 0.03 | -0.07 | -0.07 | 1.00 | 0.10 | 0.11 | 0.11 |
| Vitamin D | 0.08 | -0.08 | 0.08 | 0.10 | 1.00 | 0.04 | 0.07 |
| Calcium | 0.06 | 0.02 | 0.02 | 0.11 | 0.04 | 1.00 | 0.06 |
| Zinc | 0.08 | -0.12 | 0.02 | 0.11 | 0.07 | 0.06 | 1.00 |

*EPA, eicosapentaenoic acid; DHA, docosahexaenoic acid.*
